# Disseminated carcinomatosis of the bone marrow from pancreatic cancer: a case report

**DOI:** 10.1186/s12885-016-2849-1

**Published:** 2016-10-13

**Authors:** Hiroki Namikawa, Yasuhiko Takemoto, Ayako Makuuchi, Masanori Kobayashi, Shigeki Kinuhata, Mina Morimura, Takashi Ikebe, Hiromu Tanaka, Taichi Shuto

**Affiliations:** 1 Department of Medical Education and General Practice, Osaka City University, Graduate School of Medicine, 1-4-3, Asahi-machi, Abeno-ku, Osaka, 545-8585 Japan; 2 Department of Emergency and General Practice, Higashi Sumiyoshi Morimoto Hospital, 3-2-66, Takaai, Higashisumiyoshi-ku, Osaka, 546-0014 Japan; 3 Department of Palliative Care, Higashi Sumiyoshi Morimoto Hospital, 3-2-66, Takaai, Higashisumiyoshi-ku, Osaka, 546-0014 Japan

**Keywords:** Disseminated carcinomatosis of the bone marrow, Disseminated intravascular coagulation, Diffuse bone metastases, Pancreatic cancer

## Abstract

**Background:**

Most cases of disseminated carcinomatosis of the bone marrow (DCBM) arise from gastric cancer. DCBM from pancreatic cancer is very rare. We herein present a case of DCBM from pancreatic cancer.

**Case presentation:**

A 57-year-old man was referred to our hospital for severe lumbago. Laboratory data indicated that he suffered from disseminated intravascular coagulation (DIC). Non-contrast abdominal computed tomography (CT) revealed multiple bone masses but no other abnormal findings. Left iliac bone marrow biopsy revealed poorly differentiated adenocarcinoma cells. Positron emission tomography (PET)-CT showed diffuse abnormal uptake in the bones and tail of the pancreas. Contrast whole-body CT showed a tumor measuring approximately 28 mm in diameter with poor enhancement in the tail of the pancreas. The patient’s final diagnosis was pancreatic cancer located in the tail of the pancreas with diffuse bone metastases and DIC. His DCBM was thus believed to originate from the pancreatic cancer. He succumbed to the disease approximately 2 months after admission to our hospital.

**Conclusion:**

We herein describe a case of pancreatic cancer located in the tail of the pancreas with diffuse bone metastases and DIC, which, in our case, was DCBM. Therefore, in cases of DCBM with an unknown primary tumor, pancreatic cancer should be considered during differential diagnosis.

## Background

Disseminated carcinomatosis of the bone marrow (DCBM) caused by solid tumors is often accompanied by disseminated intravascular coagulation (DIC) [1]. The prognosis of patients with DCBM is generally very poor [2]. Approximately 80–90 % of all DCBM cases are found in stomach cancer patients. [3]. However, DCBM arising from pancreatic cancer is extremely rare. We herein report the case of a 57-year-old man who had pancreatic cancer located in the tail of the pancreas with diffuse bone metastases and DIC.

## Case presentation

### Case description

A 57-year-old man was referred to our hospital for severe lumbago. His symptoms appeared approximately 3 months prior to referral. After experiencing such symptoms for about a month, the patient consulted an orthopedist. A lumbar spinal radiography showed no significant findings. Despite treatment for pain relief, his lumbago persisted. At presentation, the patient reported no particular past medical history or known allergies. His medication included oxycodone hydrochloride hydrate, loxoprofen sodium hydrate, rebamipide, prochlorperazine maleate, and magnesium oxide. He did not smoke or consume alcohol, and worked as a designer. His mother had diabetes mellitus, hypertension, and hepatocellular carcinoma, and his sister had colon cancer. On physical examination, the patient weight 82 kg and was 174 cm tall. His blood pressure was 189/128 mmHg; his pulse was 92 beats per minute; body temperature was 36.9 °C; respiratory rate was 18 breaths per minute, and the oxygen saturation level was 98 % while breathing ambient air. No swelling of the lymph nodes was palpable. His physical examination was otherwise normal. Laboratory data showed a white blood cell count of 11,100/mm^3^, platelet count of 73,000/mm^3^, international normalized ratio for prothrombin time of 1.15, fibrinogen level of 102 mg/dL, fibrin degradation products of 65.7 μg/mL, d-dimer level of 18.1 μg/mL, antithrombin-III activity of 61 %, C reactive protein level of 3.63 mg/dL, creatinine level of 1.32 mg/dL, calcium level of 11.3 mg/dL, aspartate aminotransferase level of 164 U/L, alanine aminotransferase level of 119 U/L, alkaline phosphatase level of 547 U/L, and lactate dehydrogenase level of 552 U/L. Other test results are shown in Table 1. These laboratory data suggested that the patient had DIC.

**Table 1:** Laboratory Data

Variable	On Admission	Two Weeks after Presentaion
White blood cell count (per mm^3^)	11100	12100
Differential count (%)		
Neutrophils	73	
Lymphocytes	15	
Monocytes	9	
Eosinophils	0	
Basophils	0	
Myelocytes	3	5.0
Metamyelocyte	0	1.0
Hemoglobin (g/dL)	15.0	14.5
Hematocrit (%)	42.6	
Platelet count (per mm^3^)	73000	75000
international normalized ratio for prothrombin time	1.15	2.14
Activated partial-thromboplastin time (sec)	29.8	41.7
Fibrinogen (mg/dL)	102	150
Fibrin degradation products (μg/mL)	65.7	98.8
D-dimer (μg/mL)	18.1	34.7
Antithrombin- III activity (%)	61	
C reactive protein (mg/dL)	3.63	8.56
Total protein (g/dL)	7.1	
Albumin (g/dL)	4.2	3.8
Blood urea nitrogen (mg/dL)	27	36
Creatinine (mg/dL)	1.32	1.44
Sodium (mEq/L)	143	
Potassium (mEq/L)	3.3	
Chloride (mEq/L)	104	
Calcium (mg/dL)	11.3	
Total bilirubin (mg/dL)	1.2	
Direct bilirubin (mg/dL)	0.5	
Asparate aminotransferase (U/L)	164	
Alanine aminotransferase (U/L)	119	
Alkaline phosphatase (U/L)	547	1012
Lactate dehydrogenase (U/L)	552	608
Carcinoembryonic antigen (ng/mL)	23.6	
Carbohydrate antigen 19-9 (U/mL)	72836	
Span-1 (U/mL)	5200	
DUPAN-2 (U/mL)	440	

**Fig. 1:**
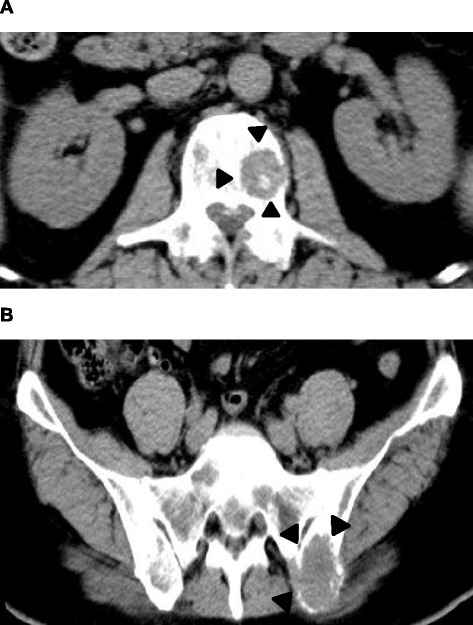
Non-contrast abdominal computed tomography (CT) images. **a**: Multiple bone masses; **b**: Multiple bone masses (*black arrowheads*) from the abdominal area

**Fig. 2:**
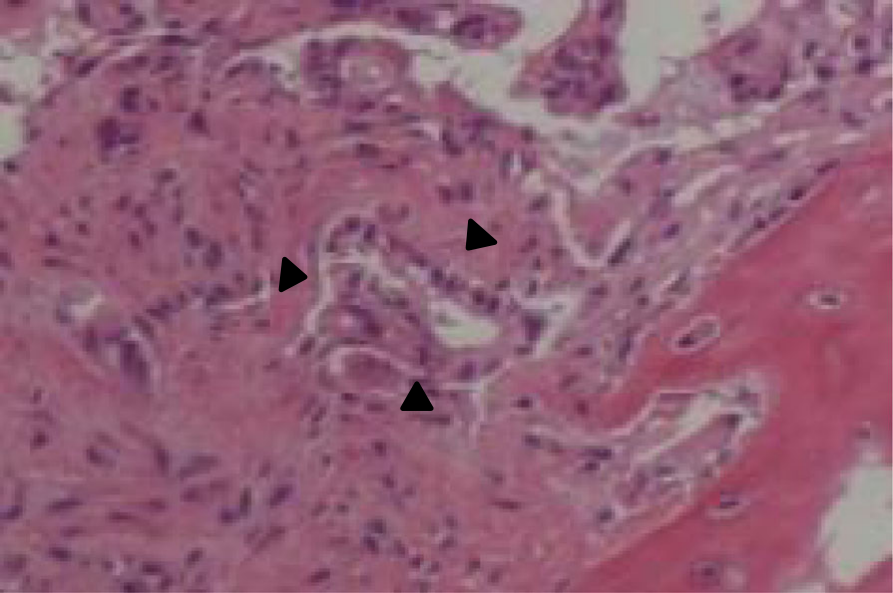
Bone marrow biopsy. Hematoxylin and eosin staining of the bone marrow biopsy performed at the left iliac revealed adenocarcinoma cells (×400, *black arrowheads*)

**Fig. 3:**
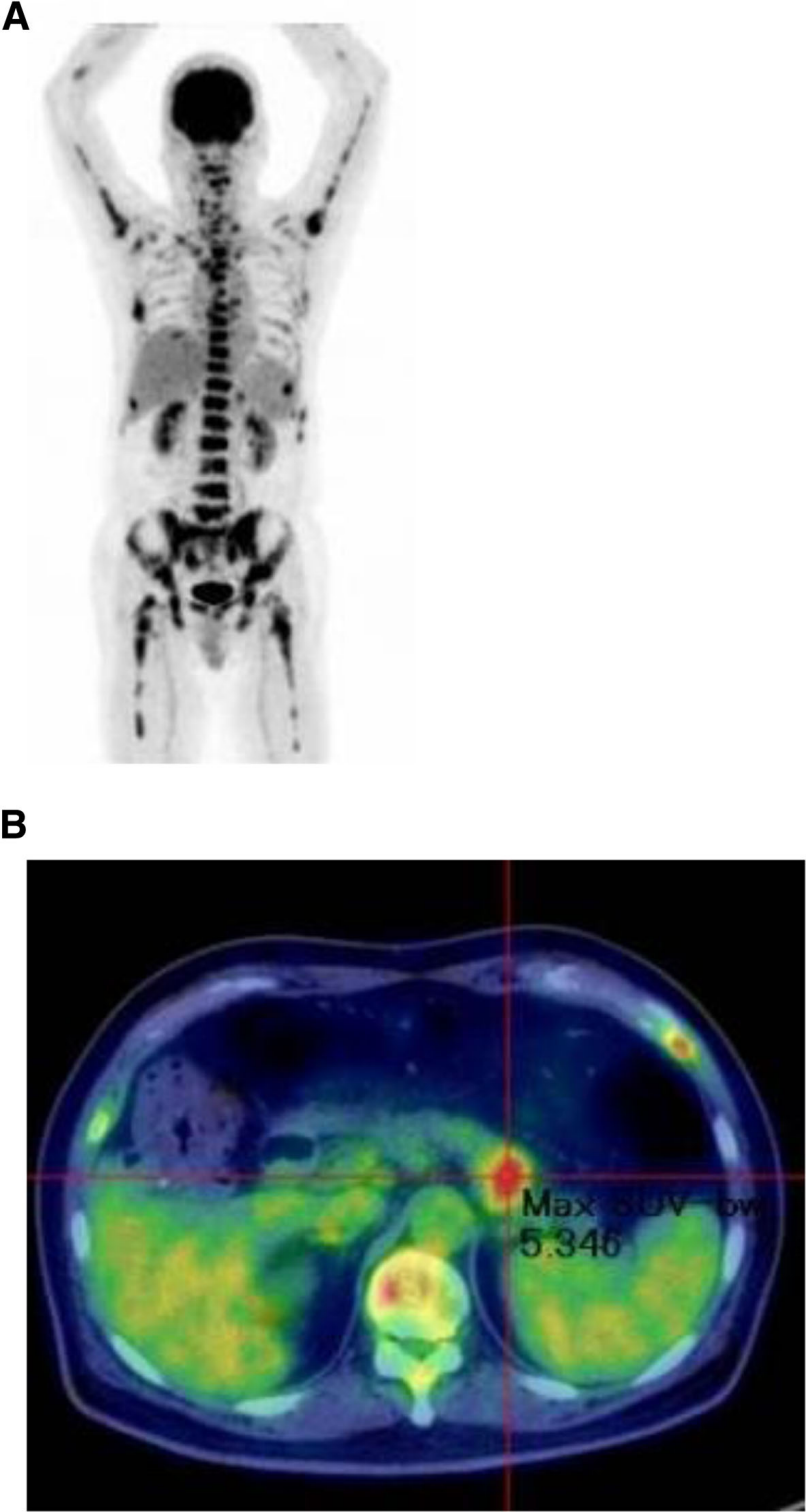
PET-CT studies. **a**: Diffuse skeletal uptake; **b**: Abnormal uptake in the tail of the pancreas

**Fig. 4:**
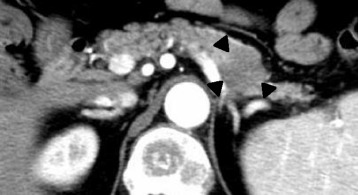
Contrast-enhanced whole-body CT image. A tumor of approximately 28 mm in diameter with poor enhancement was detected in the tail of the pancreas (*black arrowheads*)

Abdominal ultrasonography showed mild splenomegaly. Non-contrast chest computed tomography (CT), gastrointestinal endoscopy, and colonoscopy detected no abnormal findings. Non-contrast abdominal CT revealed multiple bone masses (Fig. 1); however, no tumors were found in the liver, adrenal glands, kidneys, bladder, prostate, or pancreas. Therefore, a bone marrow biopsy was performed at the left iliac, revealing poorly differentiated adenocarcinoma cells (Fig. 2). Immunohistochemical studies of the biopsy specimen showed positivity for keratin 7, carcinoembryonic antigen (CEA), and cancer antigen (CA) 19-9; negativity for prostatic acid phosphatase (PAP) and napsin A, while inconclusive for keratin 20 and thyroid transcription factor (TTF)-1. Positron emission tomography (PET)-CT disclosed diffuse abnormal uptake in the bones and tail of the pancreas (Fig. 3), and contrast enhanced whole-body CT depicted a tumor of approximately 28 mm in diameter with poor enhancement in the pancreatic tail (Fig. 4). The patient’s final diagnosis was pancreatic cancer located in the tail of the pancreas with diffuse bone metastases and DIC. His DCBM was thus believed to originate from the pancreatic cancer.

We administered nafamostat mesilate to treat DIC and opioid analgesics to relieve the patient’s lumbago and sharp osteocopic pain. However, we were unable to administer systemic chemotherapy owing to his poor condition. The patient’s general status rapidly deteriorate, and he was transferred to a palliative care unit (PCU). Laboratory data at discharge are shown in Table 1. He died at the PCU approximately 2 months after admission to our hospital.

### Discussion

A pathological condition derived from carcinoma and diffuse bone metastases with DIC was first reported by Jarcho in 1936 [4]. Hayashi et al. later defined such a pathological condition with DIC or microangiopathic hemolytic anemia as DCBM [1]. Almost all the previously published reports have shown that DCBM results from gastric cancer, colon cancer, lung cancer, and breast cancer [5]. To the best of our knowledge, only one prior report has described pancreatic cancer as the origin of DCBM [6]. In the present case, contrast whole-body CT revealed a tumor with poor enhancement in the tail of the pancreas and multiple bone masses. No other abnormal structures were identified by CT, gastrointestinal endoscopy, or colonoscopy, whereas PET-CT demonstrated diffuse abnormal skeletal uptake. Laboratory data in the above-mentioned case showed marked elevation of Span-1 and DUPAN-2 and DIC, while the left iliac bone marrow biopsy revealed adenocarcinoma cells. Such findings were probably indicative of pancreatic cancer with DCBM. The condition of our patient was very severe at admission, although his tumor size was significantly smaller than that in the previously reported case (28 mm vs. 55 mm) [6], and he died within 2 months of diagnosis. A young age and poorly differentiated carcinoma have been suggested to be associated with poor prognosis in gastric cancer cases with DCBM [1, 7]. Our patient was diagnosed at 57 years of age and had poorly differentiated carcinoma, which might explain his extremely poor prognosis despite a small pancreatic tumor size.

A previous report indicates that the tumor detection rate with non-contrast CT was 23 % when tumor size was less than 20 mm, 59 % for tumors measuring less than 40 mm, and 100 % in cases of tumors measuring more than 40 mm [8]. In the present case, the tumor size was 28 mm, and it was undetectable via non-contrast CT. It was subsequently found by PET and contrast-enhanced CT imaging.

While the serum CA19-9 levels were normal in cases of gastric cancer with DCBM [9–12], the serum CA19-9 levels were markedly elevated in cases of pancreatic cancer with DCBM in the present study as well as those in a previous study [6]. Although the mechanism for this finding is unknown, the extremely high CA 19-9 levels might be helpful in the early diagnosis of pancreatic cancer with DCBM before the histopathological examination results are obtained.

Exacerbation of DCBM occurs rapidly, and the mean survival time is 4.6 months after the onset of DCBM [9]. A proper diagnosis and timely administration of chemotherapy and treatment of DIC are crucial to prolong survival [13]. Pancreatic tumors are occasionally challenging to detect with non-contrast CT. Therefore, it is important to consider the pancreas as a possible origin of DCBM and perform contrast-enhanced CT when multiple bone metastases are observed.

## Conclusions

We herein describe a case of pancreatic cancer located in the tail of the pancreas with diffuse bone metastases and DIC, which, in our case, was DCBM. This is the first case report to demonstrate DCBM arising from the small pancreatic cancer, to the best of our knowledge. Therefore, in cases of DCBM with an unknown primary tumor, pancreatic cancer should be considered during differential diagnosis to properly identify the primary site for the timely administration of chemotherapy and DIC treatment.

## Abbreviations

CA 19-9: Carbohydrate antigen 19-9
CEA: Carcinoembryonic antigen
CT: Computed tomography
DCBM: Disseminated carcinomatosis of the bone marrow
DIC: Disseminated intravascular coagulation
PAP: Prostatic acid phosphatase
PCU: Palliative care unit
PET: Positron emission tomography
TTF-1: Thyroid transcription factor-1
